# Seasonal Dynamics of Fish Assemblages on Breakwaters and Natural Rocky Reefs in a Temperate Estuary: Consistent Assemblage Differences Driven by Sub-Adults

**DOI:** 10.1371/journal.pone.0075790

**Published:** 2013-09-26

**Authors:** Ashley M. Fowler, David J. Booth

**Affiliations:** Fish Ecology Laboratory, School of the Environment, University of Technology, Sydney, New South Wales, Australia; Leibniz Center for Tropical Marine Ecology, Germany

## Abstract

Development of infrastructure around cities is rapidly increasing the amount of artificial substrate (termed artificial reef, ‘AR’) in coastal marine habitats. However, effects of ARs on marine communities remain unknown, because it is unclear whether ARs can maintain similar communities to natural reefs. We investigated whether well-established (> 30 years old) breakwaters could consistently approximate fish assemblages on interspersed rocky reefs in a temperate estuary over 6 consecutive seasons using regular visual surveys between June 2009 (winter) and November 2010 (spring). We examined whether assemblage differences between reef types were driven by differences in juvenile recruitment, or were related to differences in older life-stages. Assemblages on both reef types were dominated by juveniles (61% of individuals) and sub-adults (34% of individuals). Seasonal fluctuations in assemblage parameters (species richness, diversity, sub-adult abundance) were similar between reef types, and levels of species diversity and assemblage composition were generally comparable. However, abundance and species richness were consistently higher (1.9-7.6 and 1.3-2.6 times, respectively) on breakwaters. These assemblage differences could not be explained by differences in juvenile recruitment, with seasonal patterns of recruitment and juvenile species found to be similar between reef types. In contrast, abundances of sub-adults were consistently higher (1.1-12 times) at breakwaters, and assemblage differences appeared to be driven by this life-stage. Our results indicate that breakwaters in temperate estuaries are capable of supporting abundant and diverse fish assemblages with similar recruitment process to natural reefs. However, breakwaters may not approximate all aspects of natural assemblage structure, with differences maintained by a single-life stage in some cases.

## Introduction

With the global population increasing, the expansion of coastal cities is putting increasing pressure on shallow marine ecosystems. Human practises, including fishing, agriculture and tourism, substantially affect coastal marine habitats and associated biological communities [1-6]. The development of coastal infrastructure and deployment of fishery-enhancement reefs are two such practices which are rapidly altering shallow marine ecosystems, through the addition of large amounts of artificial reef (AR) habitat. AR habitat is now more prevalent than natural reef in some areas [7,8].

Despite the increase in AR habitat, the effect on existing reef communities remains difficult to predict, because it is not clear whether communities that develop on ARs generally approximate those on natural reefs. If communities on ARs differ substantially, a major shift from natural to AR habitat could alter both the structure and function of coastal ecosystems. Fishes are by far the most investigated taxon on ARs, yet comparisons of assemblages with natural reefs have produced mixed results. Although ARs have typically been shown to support higher fish abundances than natural reefs [9-12], assemblage parameters (e.g. species richness, diversity) and species composition were found to be similar or different, depending on the study considered [8,12-14]. These discrepancies are likely to have resulted, at least in part, from the wide variety of types of ARs investigated and the wide range of ecosystems in which ARs were deployed. Further research is required to determine the circumstances under which ARs are capable of approximating assemblages on natural reefs.

While the ability of ARs to approximate fish assemblages on natural reefs may differ with season, the seasonal dynamics of assemblages on ARs have rarely been considered. Many studies comparing fish assemblages between ARs and natural reefs have not incorporated a seasonal factor [e.g. 15-18]; the few that did found that similarity of assemblages between the two reef types depended on the time of year [8,19,20]. If this is a general occurrence, it may partially explain the conflicting results among previous studies comparing assemblages between the two reef types, because studies conducted at different times of the year may yield different results. It also suggests that seasonally variable processes regulating population size and assemblage structure (e.g. recruitment) may differ between ARs and natural reefs. Processes including recruitment, post-settlement mortality, and migration are known to affect the population size of reef fishes [21-23], and if these processes differ between habitats for multiple species, they can affect overall assemblage structure. There has been little investigation of population-regulating processes on ARs, or how differences in these processes influence assemblage differences between ARs and natural reefs.

Breakwaters are a common structure in coastal areas and represent a large addition of AR habitat to shallow marine ecosystems. Numerous studies have examined fish assemblages on breakwaters [8,12,14,18], yet little is known about assemblages that develop on breakwaters within estuaries. Comparisons of fish assemblages between breakwaters and estuarine reefs are important for assessing the likely impact of these structures on natural estuarine communities, particularly in urbanised estuaries where breakwaters can comprise a large proportion of available reef habitat [7]. Shallow protected reefs such as those in estuaries also function as nursery grounds for many reef fishes [24,25], and juvenile and sub-adult life-stages can comprise a substantial proportion of assemblages [26,27]. Potential differences in recruitment processes between breakwaters and estuarine reefs may therefore have a disproportionately large effect on the ability of breakwaters to approximate assemblages on estuarine reefs. Lower recruitment on breakwaters could also affect the abundance of species that rely on estuarine reefs for population replenishment.

The aims of this study were therefore to: 1) determine whether breakwaters approximate fish assemblages on rocky reefs in a temperate estuary across seasons and 2) determine if differences in assemblages between reef types are driven by seasonal differences in juvenile recruitment, or differences in older life-stages. The age (> 30 years) of the breakwaters examined in this study reduced potential bias of incomplete assemblage development on comparisons with natural reefs [28,29]. Breakwaters were also interspersed with rocky reefs to avoid any spatial confounding of assemblage patterns.

## Methods

### Ethics statement

This was an observational study and did not involve capture or handling of fishes. Survey procedures were approved by the University of Technology, Sydney’s Animal Care and Ethics Committee (Permit No: 2008-016A) and conformed to guidelines of the Australian Code of Practice for the Care and Use of Animals for Scientific Purposes.

### Study location

**Figure 1:**
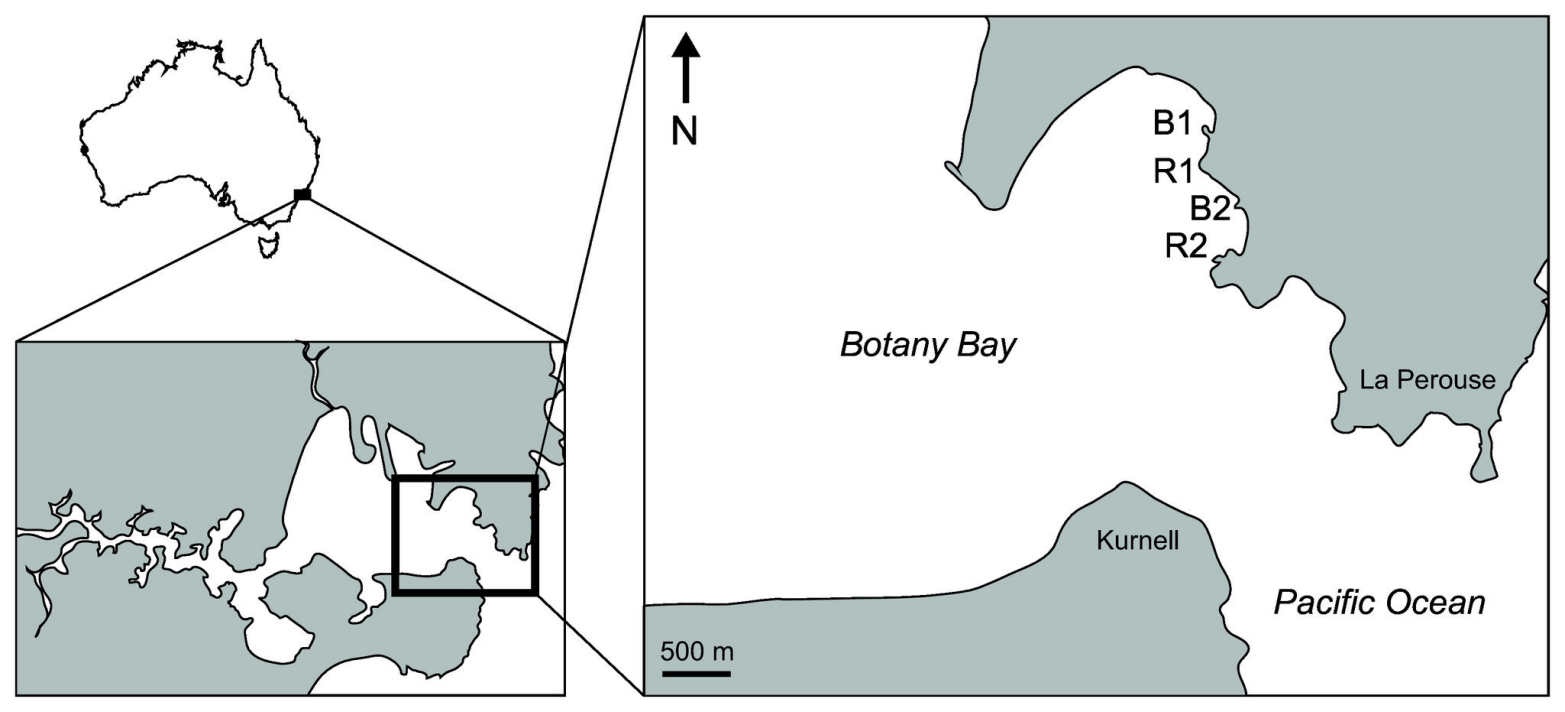
Location of breakwaters (B1, B2) and rocky reefs (R1, R2) in Botany Bay, southeastern Australia.

Fish assemblages were compared between two breakwaters and two natural rocky reefs in Botany Bay (33°59'07″ S, 151°13'38″ E), southeastern Australia, over 6 consecutive seasons between June 2009 (winter) and November 2010 (spring). Botany Bay is a sheltered embayment that forms the lower section of the Botany Bay/Georges River estuary [30]. The bay is located on the southern side of the Sydney Metropolitan area and has been extensively altered through the development of port infrastructure. Breakwaters (B1 and B2) and rocky reef sites (R1 and R2) were interspersed along the northern shoreline, 2.5-3.3 km from the coastal entrance to the Bay (Figure 1), and were separated from each other and other hard substrata by at least 100 m of sandy habitat. Breakwaters were installed during 1976-1978, were 60 m long, and consisted of either interlocked tetra-foil concrete modules (~2 m wide, breakwater B1), or rough-cut granitic blocks (~0.5-1.5 m^3^, breakwater B2). Natural rocky reef sites were located on headlands and consisted of emergent sandstone reef. Compared to breakwaters, rocky reef habitat generally sloped more gradually and was less rugose, with fewer and smaller interstices. Breakwater and rocky reef habitats only contained small amounts of attached macroalgae, but turfing algae was common on both reef types.

### Sampling method

Fish assemblages were surveyed monthly by a single experienced observer (AMF) who counted all fish along replicate 20 × 2 m belt transects using mask and snorkel. Snorkelling was a suitable survey method because depth did not exceed 3 m at any site [31]. On each sampling occasion, 2 replicate transects were surveyed at each breakwater and 4 replicate transects were surveyed at each rocky reef site. Further replication on breakwaters was not possible because large sections were ≤ 1 m deep and inclusion of these sections would likely have biased comparisons between reef types. Transects were swum once at a slow (≤ 0.1 m s^-1^) speed. Larger mobile species ahead of the observer were counted first, and holes and crevices underneath the observer were then searched for smaller cryptic species. This method was suitable for obtaining accurate abundance estimates in the current study, due to the few cryptic taxa present and the narrow dimensions of most crevices. Fish were identified to species [32] and assigned to one of 3 life-stages (juvenile, sub-adult, and adult). Assignment to life-stage was based on the individual’s size relative to the maximum size attained by the species (from [32]). When more than 50 individuals of one species were present in a school, abundances were estimated to within 10% of the approximate total (i.e. nearest 10 for ~100 individuals, nearest 50 for ~500 individuals). All sites were surveyed within 3 hours on the same day and were surveyed in a random order on each sampling occasion. All surveys were completed within 2 hours of high-tide to avoid any effect of tidal state on comparisons among sampling occasions. Surveys could not be completed in October and November in 2009, and February and May in 2010, due to large swell conditions and poor visibility.

### Data analysis

Fish assemblages were compared between breakwaters and rocky reefs using both multivariate and univariate techniques. The similarity of whole assemblages was visually examined using ordination plots generated from non-metric multi-dimensional scaling (nMDS) performed on ranked Bray-Curtis similarities (PRIMER v. 6, PRIMER-E Ltd). Abundance data were fourth-root transformed to balance the contribution of abundant and rare species to similarity values [33]. To examine the effect of season on assemblage similarities, separate ordinations were done for each season. Monthly abundance data were pooled within seasons.

Assemblage differences indicated by nMDS were tested for significance using PERMANOVA (PERMANOVA+, PRIMER-E). PERMANOVA is the equivalent of an ANOVA performed on similarity values and uses permutations to test the significance of differences among groups [34]. It is preferred to other multivariate analyses (e.g ANOSIM) for multi-factor designs because it provides a test for interactions among factors. A repeated-measures design was used with three factors: reef type (2 levels: breakwaters and rocky reefs), season (6 levels: winter 2009-spring 2010), and site (4 levels). Reef type and season were treated as fixed, while site was treated as random and nested within reef type. Differences between reef types were tested using Monte-Carlo p-values, because not enough unique permutations were possible to determine permutational p-values for this factor. Monte-Carlo p-values provide an approximation of significance based on asymptotic theory and should be used in preference to the permutational p-values when the number of unique permutations is < 999 [34]. When significant differences were found, pair-wise PERMANOVA tests were used to determine differences between levels within a factor. The variability of assemblages was also compared between reef types using the PERMDISP procedure, which is the multivariate equivalent of Levene’s test for homogeneity of variance in ANOVA [34].

To determine if fish abundances and assemblage parameters differed between breakwaters and rocky reefs, total abundance (number of individuals per 10 m^2^), species richness (*S*), and diversity (Shannon-Wiener, *H*'), were compared between the two reef types using univariate PERMANOVAs. Univariate PERMANOVAs were also used to compare the abundances of different life-stages (juveniles, sub-adults, adults) between breakwaters and rocky reefs. In all cases, the same three-factor design as the multivariate analysis was used. PERMANOVAs were used instead of parametric alternatives because data for most variables were highly skewed and transformation did not correct non-normality. Monte-Carlo p-values were used for pair-wise comparisons due to the low number of unique permutations possible. For analyses of juvenile abundance, only sampling occasions at least 2 months apart were included to reduce the effect of repeated sampling of the same individuals on recruitment estimates. Data were log_10_(x+1) transformed to meet the assumption of homogeneity of dispersion when required. A p-value < 0.05 was considered significant for all tests.

## Results

**Figure 2:**
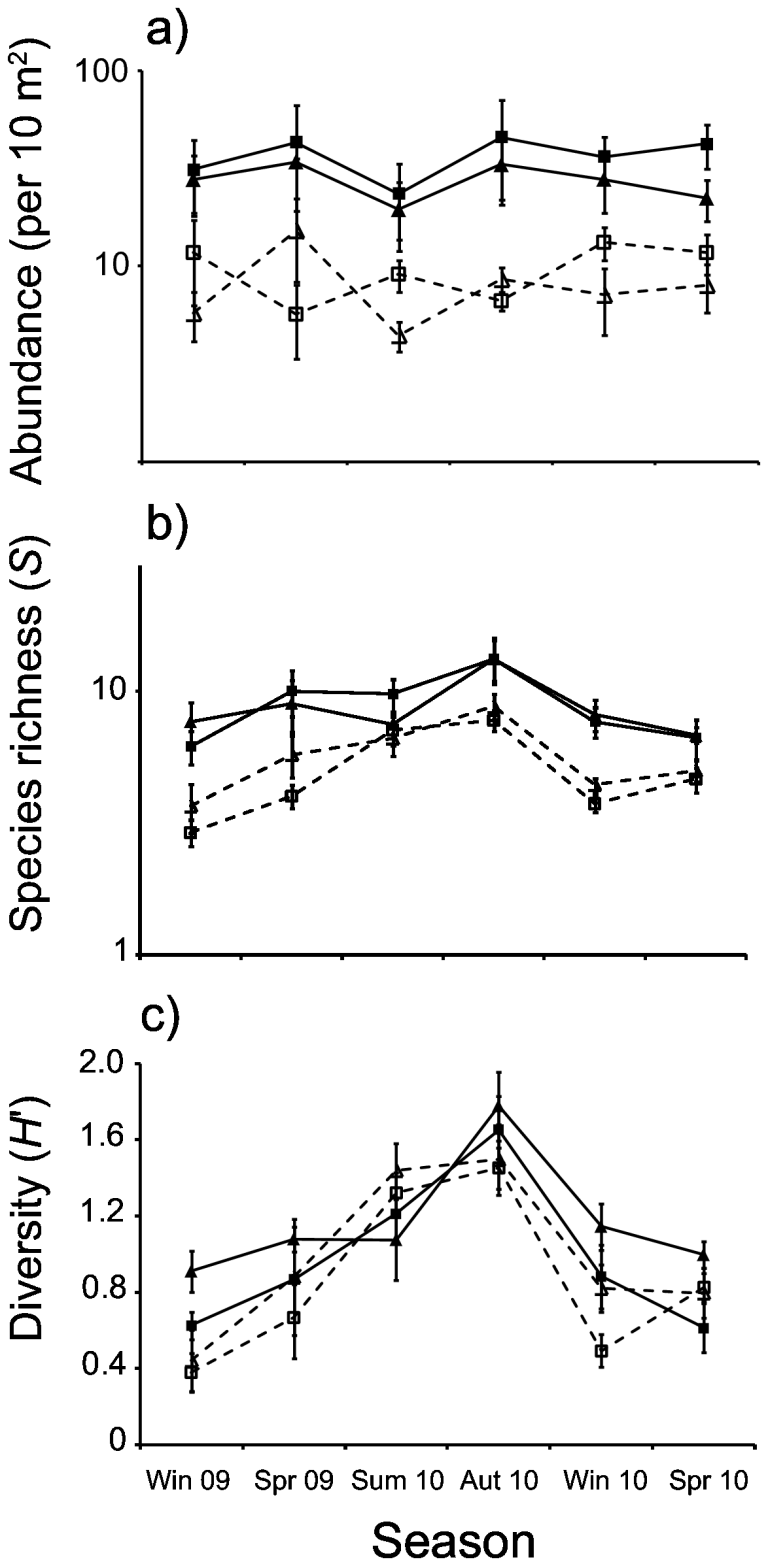
Assemblage parameters on breakwaters (solid lines; square = B1, triangle = B2) and rocky reefs (dashed lines; square = R1, triangle = R2) during 6 consecutive seasons. Mean a) abundance, b) species richness, and c) Shannon Wiener diversity are displayed. Bars indicate standard errors. Note log_10_ scale of y-axes axes for both abundance and species richness.

Overall, 10 870 individuals of 39 species were recorded in the study area (Table 1). Assemblage data are provided in Table S1 to allow further use of this comprehensive dataset. Total fish abundance was higher (seasonal range: 1.9-7.6 times) on breakwaters than at rocky reefs (Figure 2a; PERMANOVA, *F*_1,3_ = 113.2, p = 0.002), and a lack of interaction between reef type and season indicated this difference was consistent across seasons (PERMANOVA, *F*_5,14_ = 0.507, p = 0.75). A significant effect of season on total abundance on both reef types was also found (PERMANOVA, *F*_5,14_ = 3.171, p = 0.04), however a seasonal pattern (e.g. lower abundances in winter and higher abundances in summer) was not evident at either reef type (Figure 2a).

**Table 1:** Species of fish observed on breakwaters and rocky reefs during the study.

**Family**	**Species**	**Family**	**Species**
Atherinidae	* Atherinomorus vaigiensis *	Aplodactylidae	* Aplodactylus lophodon *
Tetrarogidae	* Centropogon australis *	Cheilodactylidae	* Cheilodactylus fuscus *
Platycephalidae	* Platycephalus fuscus *		* Cheilodactylus vestitus *
Ambassidae	* Ambassis jacksoniensis *	Pomacentridae	* Abudefduf bengalensis *
Plesiopidae	* Trachinops taeniatus *		* Abudefduf sexfasciatus *
Dinolestidae	* Dinolestes lewini *		* Abudefduf vaigiensis *
Carangidae	* Trachurus novaezelandiae *		* Chromis hypsilepis *
Gerreidae	* Gerres subfasciatus *		* Parma microlepis *
Sparidae	* Acanthopagrus australis *	Labridae	* Achoerodus viridis *
Mullidae	* Parupeneus spilurus *		* Eupetrichthys angustipes *
Pempheridae	* Pempheris compressa *		* Notolabrus gymnogenis *
Kyphosidae	* Atypichthys strigatus *		* Pictilabrus laticlavius *
	* Girella elevata *		* Pseudolabrus guentheri *
	* Girella tricuspidata *	Acanthuridae	* Prionurus microlepidotus *
	* Kyphosus sydneyanus *	Monacanthidae	* Brachaluteres jacksonianus *
	* Microcanthus strigatus *	Aracanidae	* Anoplocapros inermis *
	* Scorpis lineolata *	Tetraodontidae	* Tetractenos glaber *
Monodactylidae	* Schuettea scalaripinnis *		* Torquigener pleurogramma *
Enoplosidae	* Enoplosus armatus *	Diodontidae	* Dicotylichthys punctulatus *
Chironemidae	* Chironemus marmoratus *		

Similar seasonal patterns of species richness and Shannon Wiener diversity were observed at both breakwaters and rocky reefs, with lower values in winter/spring and higher values in summer/autumn (Figure 2b, 2c). However, significant interactions between reef type and season indicated differences in both variables between reef types in certain seasons (Species richness: PERMANOVA, *F*_5,18_ = 4.969, p = 0.016; Diversity: PERMANOVA, *F*_5,15_ = 3.887, p = 0.032). Species richness was higher (1.3-2.6 times) at breakwaters than at rocky reefs in winter 2009, autumn 2010, winter 2010, and spring 2010 (Figure 2b; pair-wise PERMANOVA, p < 0.02 for all), but did not differ between the two reef types in spring 2009 and summer 2010 (p = 0.06 and 0.13, respectively). Diversity was similar between the two reef types in all seasons except autumn 2010 (Figure 2c, pair-wise PERMANOVA, p = 0.02).

**Figure 3:**
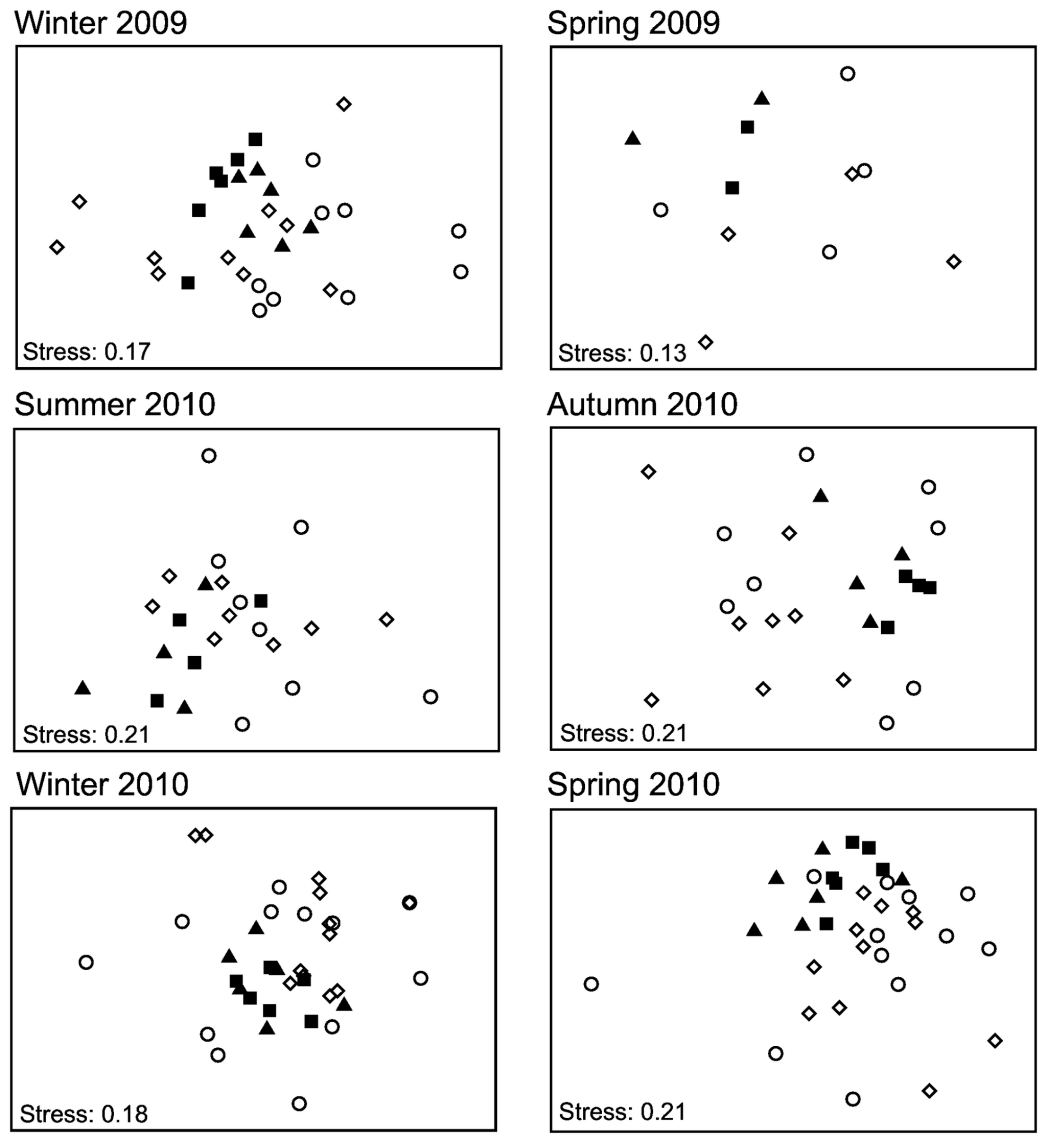
Assemblage composition on breakwaters and rocky reefs during 6 consecutive seasons. Graphs are the result of nMDS ordinations comparing multivariate fish assemblage data between breakwater (closed symbols; triangle = B1, square = B2) and rocky reef (open symbols; diamond = R1, circle = R2) transects within each season.

Multivariate PERMANOVA identified significant assemblage differences between breakwaters and rocky reefs (*F*_1,2_ = 4.896, p = 0.006), and these differences were consistent across seasons (reef type × season, *F*_5,12_ = 1.076, p = 0.38). PERMANOVA is sensitive to both separation and dispersion (variability) of multivariate data groups, and PERMDISP identified significant differences in variability between breakwaters and rocky reefs (*F*_1,161_ = 44.52, p = 0.0001). The significant PERMANOVA result may therefore have resulted from differences in both assemblage composition and variability between reef types, or solely from differences in variability [34]. Ordinations were conducted within each season to visually identify any compositional differences (separation of data groups) between reef types without the potential obscuring effect of seasonal variability. This revealed only minor compositional differences between breakwaters and rocky reefs within each season (Figure 3). Overall, only 4 species were unique to each reef type, and all except *Pseudolabrus* *guentheri* (Labridae: only found at breakwaters) were rare (≤ 5 individuals across all samples). The limited separation of data groups between reef types indicated assemblage differences identified by PERMANOVA were primarily the result of greater assemblage variability at rocky reefs compared to breakwaters. Differences in assemblage variability were spatial, rather than temporal, because rocky reef data was more variable than breakwater data within each season (Figure 3).

**Figure 4:**
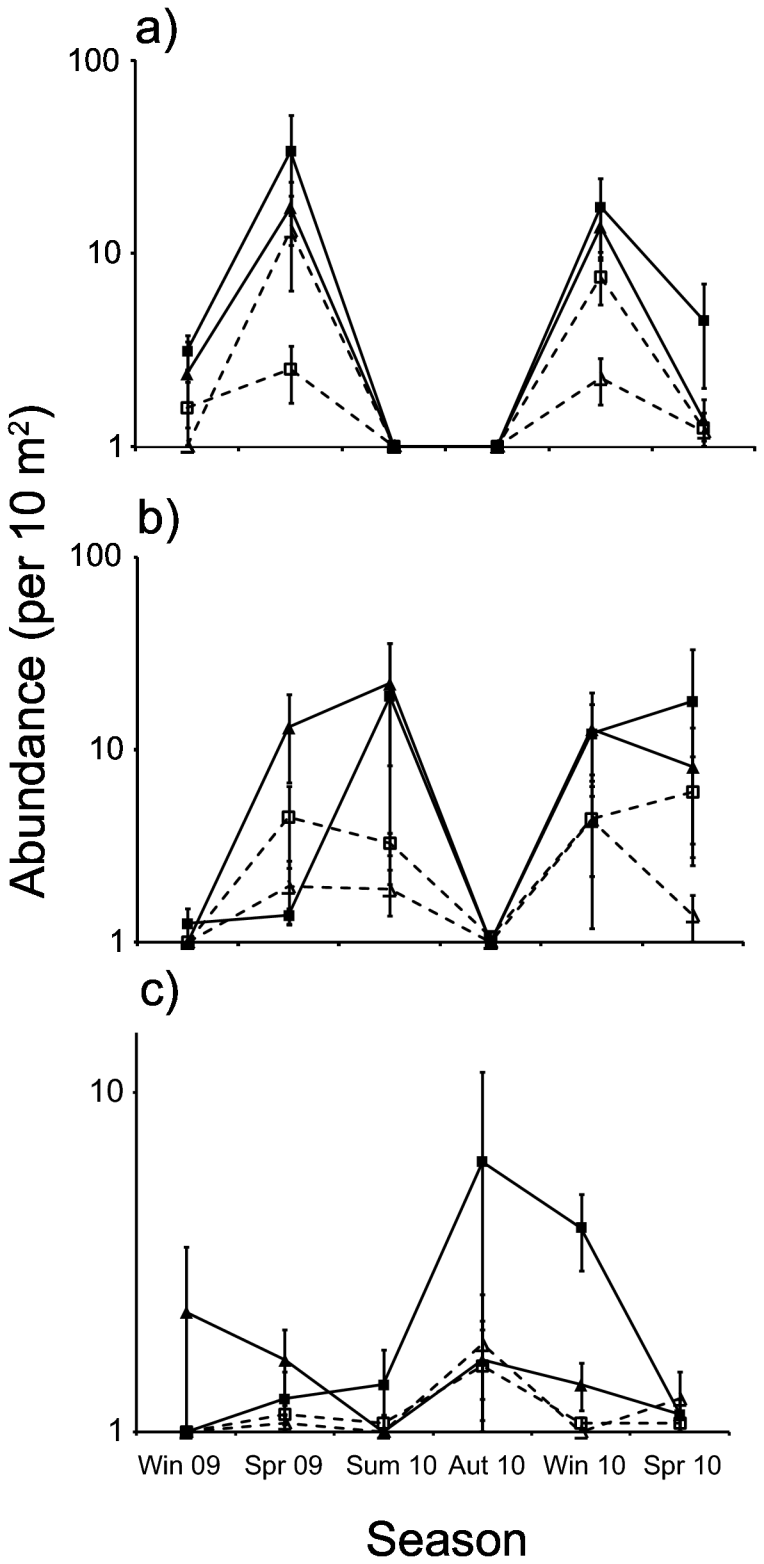
Juvenile abundances on breakwaters (solid lines; square = B1, triangle = B2) and rocky reefs (dashed lines; square = R1, triangle = R2) during 6 consecutive seasons. Mean juvenile abundance of a) *Scorpis* *lineolata*, b) *Atypichthys* *strigatus*, and c) combined remaining species are displayed. Bars indicate standard errors. Note log_10_ scale of all y-axes.

Assemblages were primarily composed of juvenile (61% of total fish) and sub-adult (34%) life-stages. Juveniles of 11 species were recorded in the study area, 8 on breakwaters and 9 on rocky reefs. However, *Scorpis* *lineolata* and *Atypichthysstrigatus* together composed 95% of individuals, so patterns of juvenile abundance for these species were analysed separately. Strong seasonal patterns of juvenile abundance were found for both species (Figure 4a, 4b; *S* *. lineolata* : PERMANOVA, *F*_5,12_ = 17.02, p = 0.0006; *A*. *strigatus*: PERMANOVA, *F*_5,13_ = 11.74, p = 0.002), with peak abundances occurring in winter and spring for *S* *. lineolata* and winter, spring, and summer for *A*. *strigatus*. Juvenile *S* *. lineolata* were 1.3-19 times (range of means across seasons) more abundant at breakwaters than at rocky reefs during seasons of recruitment (Figure 4a; PERMANOVA, *F*_1,3_ = 24.18, p = 0.02). An apparently similar pattern of higher juvenile abundance on breakwaters compared to rocky reefs was not significant for *A*. *strigatus* (Figure 4b; PERMANOVA, *F*_1,2_ = 6.369, p = 0.12). When *A*. *strigatus* and *S* *. lineolata* were excluded, the abundance of remaining juveniles was still found to differ significantly between breakwaters and rocky reefs (PERMANOVA, *F*_1,4_ = 40.50, p = 0.004). However, this result was influenced by significantly higher variability of juvenile abundance at breakwaters compared to rocky reefs (PERMDISP, *F*_1,81_ = 21.07, p = 0.002), and graphical inspection did not indicate a consistent difference in mean abundance between reef types (Figure 4c). Remaining juveniles were composed primarily of *Schuetteascalaripinnis* (54%), *Pempheris* *compressa* (15%), *Chironemus* *marmoratus* (10%), *Cheilodactylus* *fuscus* (8%), *Microcanthus* *strigatus* (7%), and *Parupeneus* *spilurus* (5%). The lack of significant interactions between reef type and season for *S* *. lineolata* (PERMANOVA, *F*_5,12_ = 1.701, p = 0.24), *A*. *strigatus* (PERMANOVA, *F*_5,13_ = 2.357, p = 0.13), and remaining juveniles (PERMANOVA, *F*_5,12_ = 1.357, p = 0.33), indicated that seasonal patterns of recruitment were similar between breakwaters and rocky reefs.

**Figure 5:**
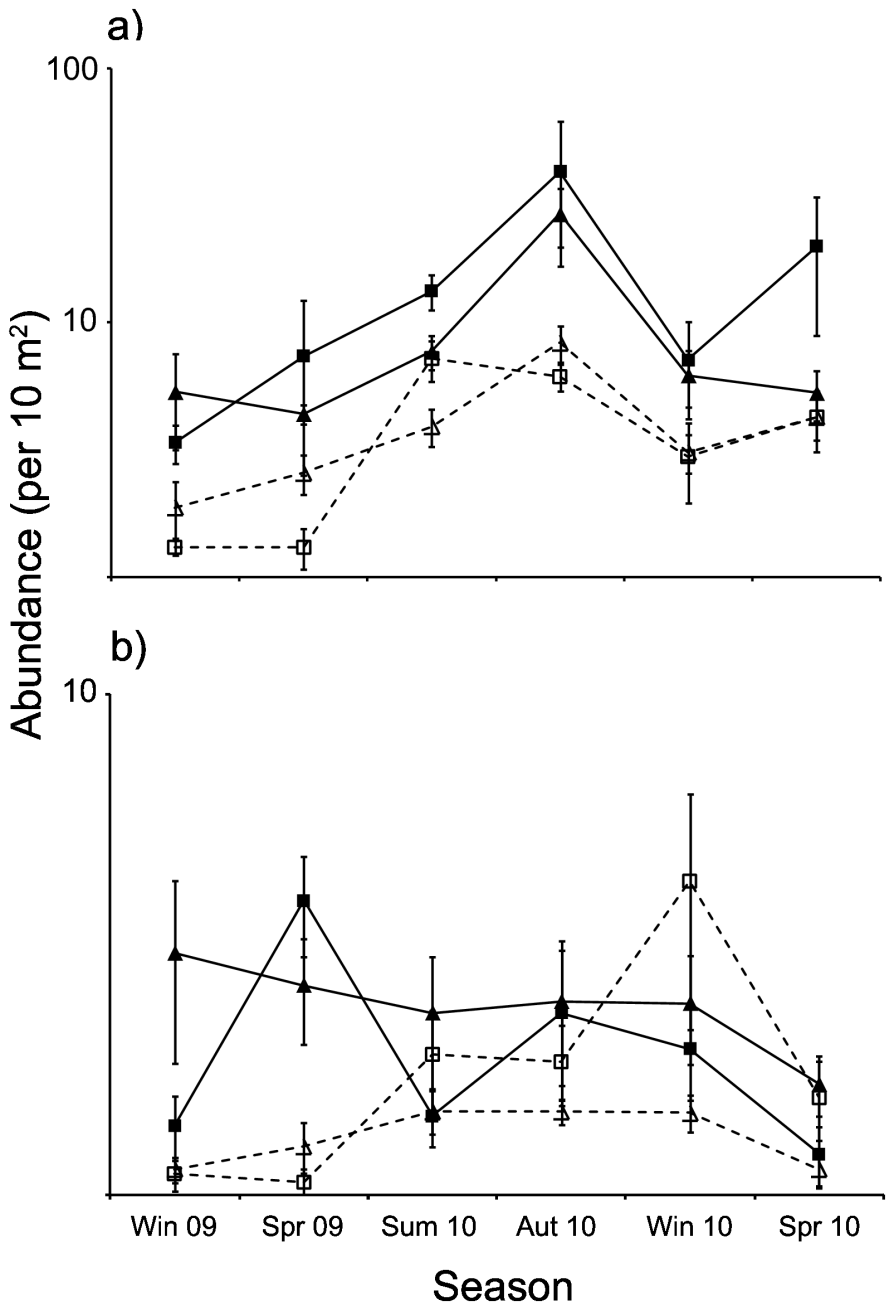
Sub-adult (a) and adult abundances (b) on breakwaters (solid lines; square = B1, triangle = B2) and rocky reefs (dashed lines; square = R1, triangle = R2) during 6 consecutive seasons. Bars indicate standard errors. Note log_10_ scale of both y-axes.

Sub-adults represented 36 of the 39 species recorded in the study area, and total abundance of this group was consistently higher (seasonal range: 1.1-12 times) at breakwaters than at rocky reefs (Figure 5a; PERMANOVA, *F*_1,3_ = 54.71, p = 0.009). Seasonal patterns of abundance were similar to patterns observed for species richness and diversity (Figure 2b, 2c), and did not differ significantly between the two reef types (PERMANOVA, reef type × season, *F*_5,12_ = 0.488, p = 0.76). Adults comprised 25 species, but only accounted for 5% of individuals recorded in the study area. Adult abundance did not differ between breakwaters and rocky reefs (Figure 5b; PERMANOVA, *F*_1,2_ = 13.04, p = 0.06), and although seasonal patterns of abundance appeared to differ between the two reef types, the interaction between reef type and season was not significant (PERMANOVA, *F*_5,13_ = 2.828, p = 0.06).

## Discussion

Urban development is rapidly increasing the amount of artificial reef (AR) habitat in marine ecosystems, yet the potential effects of this disturbance on natural communities are not well understood. An important knowledge gap concerns the ability of AR habitat to approximate communities on natural reefs. If AR habitat is unable to develop and support similar communities to natural reefs, a major shift from natural to AR habitat could alter the structure and function of existing reef communities. Such habitat shifts are already occurring in heavily-urbanised areas where, in some cases, AR habitat provided by infrastructure (e.g. breakwaters, seawalls) is now more prevalent than natural reef habitat [7,8]. We assessed the ability of breakwaters to approximate fish assemblages on natural rocky reefs over 6 consecutive seasons in a temperate estuary, an ecosystem type that has received little attention regarding the performance of AR habitat. We also examined whether differences in assemblages between reef types were driven by seasonal differences in juvenile recruitment, or differences in older life-stages.

### Approximation of rocky reef assemblages

Breakwaters were found to broadly approximate the fish assemblages on nearby rocky reefs in the study estuary (Botany Bay). Levels of species diversity and assemblage composition were similar between the two reef types, as were seasonal fluctuations in assemblage parameters (species richness, diversity, sub-adult abundance) over the 6 consecutive seasons investigated. These findings suggest that large-scale replacement of rocky reef habitat with breakwaters may not dramatically alter the structure or composition of reef fish assemblages in the study estuary. However, higher abundances and species richness found on breakwaters indicate that breakwaters did not mimic all aspects of assemblage structure on rocky reefs. Replacement of rocky reef habitat with breakwaters may therefore result in some changes to existing assemblages. Assemblages in Botany Bay may have already been altered to some degree, given that approximately 20% of the shoreline now consists of breakwaters, seawalls, and wharves [35].

Most investigations of breakwater assemblages have been conducted in open coastal areas, and consequently, little is known about the ability of breakwaters to approximate assemblages on reefs in estuaries. Results from the open coast are not necessarily indicative of the performance of breakwaters in estuaries, because coastal and estuarine fish assemblages usually differ considerably in species composition, size-, and age-structure [27,36]. In support, we found breakwater assemblages were dominated by juvenile and sub-adult stages, a result which has not been reported from investigations in coastal areas. To our knowledge only two previous investigations have compared fish assemblages between breakwaters and estuarine reefs [11,35]. Our results are similar to those of Lincoln-Smith et al. [11], who found breakwaters supported consistently higher abundances and species richness than rocky reefs in another temperate estuary in southeastern Australia. Although Burchmore et al. [35] found lower abundances and species richness on a breakwater compared to a rocky reef in the same estuary as the current study (Botany Bay), this likely resulted from spatial confounding of the two reef types. Their breakwater site was located ~3 km further from the estuary mouth than the natural reef site, and likely differed in numerous characteristics that were unrelated to the habitat provided by the breakwater (e.g. supply of recruits, local productivity). Our results and those of Lincoln-Smith et al. [11] are similar to findings on the open coast [8,18,37], and suggest that breakwaters may be generally capable of supporting abundant and species-rich fish assemblages, despite failing to completely mimic assemblage parameters on natural reefs. However, further investigation is required to confirm findings in estuaries, given that investigations to date have been restricted to temperate estuaries in Australia.

### Seasonal patterns of recruitment and abundances of older life-stages

Previous investigations comparing fish assemblages between ARs and natural reefs over multiple seasons found that assemblage similarity depended on the time of year, with differences in the processes of recruitment and adult migration driving seasonal differences in assemblages [8,19,20]. In contrast, we found that differences (and similarities) between assemblages on breakwaters and rocky reefs were generally consistent across seasons. Total fish abundance was higher on breakwaters in all seasons, and despite a significant interaction between reef type and season, species richness was higher on breakwaters than rocky reefs in most seasons. Further, species composition was consistently similar between reef types, and diversity differed in only one season. We found no evidence to suggest that differences in juvenile recruitment drove assemblage differences between the two reef types, despite assemblages being dominated by juvenile stages (61% of total fish). Although juveniles of one species ( *S* *. lineolata* ) were considerably more abundant on breakwaters, recruitment patterns were strongly seasonal, and differences in recruitment of this species therefore cannot explain the consistently higher total fish abundances found at breakwaters across all seasons. Differences in the abundance of this one species also cannot explain higher species richness found on breakwaters. Overall, juvenile recruitment on breakwaters was remarkably similar to rocky reefs, with 7 of the 8 most abundant recruiting species common to both reef types. Given the similarity in recruiting species, and the fact that juvenile abundances were either similar or higher on breakwaters compared to rocky reefs, further addition of breakwater habitat in the study estuary may not strongly affect processes of reef fish recruitment.

The seasonally-consistent assemblage differences found between breakwaters and rocky reefs appeared to be related to sub-adult fish. In contrast to juveniles, the abundance of sub-adults was consistently higher at breakwaters than rocky reefs, and can therefore account for consistently higher total fish abundances found at breakwaters across seasons. Seasonal patterns of sub-adult abundance also mirrored patterns of species richness in the study area (Figures 5a, 2b), indicating sub-adult assemblages may have regulated species richness on both reef types. Sub-adults were also represented by nearly all species recorded in the study area (36 out of 39), and therefore had the potential to influence species richness. Results here support previous findings that particular life-stages are capable of causing differences in assemblage structure between ARs and natural reefs [8,19,20]. However, in contrast to previous investigations which only found seasonal effects, our findings indicate that particular life-stages are capable of maintaining assemblage differences between ARs and natural reefs over numerous seasons. ARs may need to approximate the habitat requirements of multiple life-stages (i.e. not just adults) in order to closely approximate the structure and composition of reef fish assemblages.

### Processes potentially driving assemblage differences between reef types

Higher fish abundances and species richness on breakwaters compared to rocky reefs in the current investigation likely resulted from differences in the habitat provided by the two reef types. Breakwater habitat was more vertically-oriented and more rugose, with larger and more interstices, than rocky reef habitat (AMF pers. obs.). Reefs with more interstices have been found to support higher fish abundances than those with less interstices [38,39], likely due to the greater provision of shelter space for prey species [38]. Provision of more shelter space may explain the consistently higher abundance of sub-adults found on breakwaters, as well as the higher abundance of *S* *. lineolata* juveniles. The abundance of adult (large) fish was not different between the two reef types, suggesting the number of piscivorous predators may have been similar. Therefore, more shelter space on breakwaters combined with a similar number of predators may have resulted in reduced predation relative to rocky reefs. Reduced predator efficiency on ARs as a result of greater shelter space has previously been suggested as a mechanism resulting in higher fish abundances relative to natural reefs [40,41]. If breakwaters in the current study increase survival of juveniles and sub-adults by mediating predation, they may serve to increase the size of local fish populations (i.e. fish ‘production’, see 41), providing breakwaters represent a substantial proportion of available reef habitat. Given the potential importance of increased production for conservation of fish populations, future comparisons of predator-induced mortality rates between breakwaters and natural reefs are warranted.

Higher species richness on breakwaters may have resulted from greater habitat complexity (i.e. greater rugosity, more interstices; outlined above) compared to rocky reefs. Numerous investigations have observed higher species richness of fish on reefs with greater habitat complexity [42-44], likely due to the provision of a wider range of microhabitats. Differences in habitat complexity between breakwaters and rocky reefs would also have been expected to affect species composition, due to species-specific microhabitat preferences [45], or differences in competition and predation relating to the availability and size of refuges [38,46], or both. Yet only 4 of the 39 species observed in the study areas were unique to each reef type, and all but one of these species ( *Pseudolabrus* *guentheri* ) were rare (≤ 5 individuals across all surveys). This result is contrary to many previous investigations which have found strong differences in species composition between ARs and natural reefs [8,10,13,16,29,47-49]. A possible explanation for our result may involve differences in the spatial scale of habitat complexity between the two reef types. While breakwater habitat appeared more structurally complex and provided multiple microhabitats at the scale of individual transects (20 m), rocky reef habitat was more spatially variable and provided a range of microhabitats among transects at the scale of sites (~ 100 m). Overall similarity in species composition between the two reef types may therefore have resulted from provision of a similar range of microhabitats at the scale of sites. This conclusion is supported by the greater assemblage variability observed among transects at rocky reefs.

Although we cannot confirm structural habitat differences as the cause of differences in fish assemblages between breakwaters and rocky reefs, we are able to exclude factors which vary predictably in space (e.g. wave exposure, current regime), due to the spatial interspersion of reef types in the study area (Figure 1). Food availability is therefore unlikely to explain differences in the abundance of planktivorous species, including the two most abundant species *A*. *strigatus* and *S* *. lineolata* , due to presumably similar delivery of plankton to both reef types (see 49). However, we cannot rule out potential differences in benthic food resources between breakwaters and rocky reefs. Numerous fish species recorded in the study area are benthic invertivores (e.g. labrids), and their relative abundance may have been influenced by differences in the availability of invertebrate prey. Similarly, the relative abundance of herbivorous fishes (e.g. *Girella* spp.) may have been influenced by differences in algal assemblages between reef types. Differences in macroalgal cover may have further contributed to differences in habitat complexity, despite the apparently small amount of macroalgae observed on both reef types (see methods). The different substrates provided by the two breakwaters (concrete and granite, see methods) may also have been expected to influence the development of algal and invertebrate assemblages [50], potentially generating differences in fish assemblages between the two replicate units. However, any resulting differences in fish assemblages between replicate breakwaters appeared overshadowed by differences between breakwaters and natural reefs. Despite the many comparisons of fish assemblages between ARs and natural reefs, few investigations have elucidated the causal mechanisms of assemblage differences. Further investigation of the factors responsible for differences in fish assemblages between ARs and natural reefs is required in order to minimise the installation of structures which are likely to develop assemblages that differ from existing reefs.

## Conclusions

As marine ecosystems are increasingly altered by coastal development, understanding the effects of AR habitat on marine communities is becoming critically important. From results reported here and elsewhere [8-10,15,49], it is clear that even large well-established ARs may never completely mimic the structure of fish assemblages on natural reefs, regardless of whether they are purpose-built or provide unintended habitat (i.e. infrastructure). Similar results have been found for tropical corals [8,51], and algae and invertebrates in temperate intertidal habitats [52,53]. However, the ecological importance of such assemblage differences remains unknown. For example, does it matter that fish assemblages on breakwaters differ slightly to those on natural reefs if their abundance and species richness are higher? Research to date has primarily focused on the ability of ARs to approximate community structure and composition, yet the ability of ARs to function similarly to natural reefs may be of considerably greater importance. We have shown here that breakwaters are capable of functioning similarly to estuarine reefs as recruitment habitat for juvenile fishes, but further investigation is required to assess other functional capabilities of coastal structures, including their ability to provide adequate food resources for inhabiting communities, their ability to develop reproductively-effective populations, and their ability to approximate trophic dynamics on natural reefs. The potential for ARs to facilitate biological invasions must also be considered, particularly when ARs are to be installed in close proximity to natural reefs, like the breakwaters in the current study. Common coastal structures including wharf pilings and pontoons have been found to support higher proportions of nonindigenous species (NIS) than nearby rocky reefs [54], suggesting they could act as ‘beachheads’ for invasion. Until such aspects of AR performance are fully elucidated, assessment of the effects of expanding coastal infrastructure on marine communities is likely to be limited.

## Supporting Information

Table S1 **Data used for multivariate comparisons of fish assemblages between seasons, reef types and sites.** Values are numbers of individuals per transect.
(XLSX)
